# How does artificial intelligence literacy affect university students’ innovative behavior? A serial mediation analysis based on latent profiles

**DOI:** 10.3389/fpsyg.2026.1844997

**Published:** 2026-06-11

**Authors:** Lei Ma, Tianxing Liu, Chuyuan Zhao, Tiezhu Niu

**Affiliations:** 1 Physical Education Department, Dalian Minzu University, Dalian, China; 2 School of Physical Education and Sport, Henan University, Kaifeng, China

**Keywords:** artificial intelligence anxiety, artificial intelligence literacy, cognitive flexibility, innovative behavior, latent profile analysis, serial mediation

## Abstract

**Purpose:**

Against the backdrop of the rapid proliferation of generative artificial intelligence (AI), how university students can effectively leverage AI technologies to foster their innovative behavior has emerged as a critical issue in higher education. This study aims to explore the relationship and underlying mechanisms between AI literacy and university students’ innovative behavior.

**Methods:**

A cross-sectional survey was conducted among 1,040 university students in China. Data were collected using the AI Literacy (AIL) Scale, AI Anxiety (AIA) Scale, Cognitive Flexibility (CF) Scale, and Innovative Behavior (IB) Scale.

**Results:**

AIL was significantly and positively correlated with CF (*r* = 0.346, *p* < 0.001) and IB (*r* = 0.329, *p* < 0.001), and significantly negatively correlated with AIA (*r* = −0.319, *p* < 0.001). Mediation analysis revealed three significant indirect pathways: the independent mediating effect of AIA accounted for 13.44% of the total effect, the independent mediating effect of CF accounted for 14.15%, and their serial mediating effect accounted for 3.30%. Additionally, latent profile analysis identified three latent profiles of AI literacy: low AI literacy (15.29%), moderate AI literacy (37.02%), and high AI literacy (47.69%). Significant differences were observed in the serial mediation pathways across these distinct profiles.

**Conclusion:**

AIL was directly associated with university students’ IB and showed indirect associations through the independent and serial mediation of AIA and CF. These findings extend the application of affordance theory in human-AI collaboration contexts and provide empirical evidence for higher education institutions to implement differentiated AI education and innovation cultivation strategies.

## 1 Introduction

Innovative behavior (IB) plays an increasingly critical role in university students’ career development and personal achievement, and fostering it has thus remained a core mission of higher education (Luo et al., 2025). Amabile (1988) originally defined IB as the intentional introduction of new ideas, processes, or problem-solving methods within work or study contexts. Encompassing both the generation and implementation of creative ideas, IB requires not only individual creativity but also specific knowledge and skills (Mainemelis, 2010). The rapid advancement of generative artificial intelligence (AI), particularly large language models such as ChatGPT, DeepSeek, and Gemini, is profoundly reshaping how students learn and acquire knowledge, presenting both opportunities and challenges for cultivating their IB (Zhang, 2026). According to Microsoft’s global AI adoption report, the worldwide AI penetration rate reached 16.3% in the second half of 2025, with one in six individuals using generative AI tools (Organization, 2025). On the one hand, generative AI markedly enhances learning efficiency, freeing cognitive resources for deeper thinking and innovative practices (Chen and Xiao, 2025; Li H. et al., 2026a). On the other hand, its inherent tendency to “hallucinate”—fabricating false information or fictitious sources—can disrupt students’ information screening and knowledge construction, potentially threatening the positive development of their creativity (Pavone, 2025; Zhang and Cao, 2025; Li H. et al., 2026a). How to harness AI’s technological advantages while mitigating its risks, thereby fostering university students’ creativity, has thus become a pressing concern for higher education. This concern resonates internationally. UNESCO’s *Beijing Consensus on Artificial Intelligence and Education* identifies AI literacy (AIL) as a critical competency for future educational transformation and for shaping innovators suited to the intelligent era (UNESCO, 2019). Similarly, Singapore’s Ministry of Education has embedded AI skills and literacy into its EdTech Masterplan 2030 to equip students with the core competencies needed for continuous innovation in a digitalized world (Organization, 2026). Underlying this policy consensus is a fundamental premise: as the primary users of AI technology, whether university students possess a high level of AIL directly determines their ability to translate technological potential into innovative momentum (Lu et al., 2025). Students with higher AIL can use AI tools more appropriately, confidently, and ethically, thereby expanding their cognitive boundaries, stimulating creative thinking, and translating technological advantages into tangible innovative outcomes (Zhang and Kang, 2026). Conversely, those with insufficient AIL are more vulnerable to information misjudgment, technological over-reliance, and cognitive fixation; they struggle to benefit from AI and may even fall into cognitive traps through blind trust, harming their IB (Chun et al., 2025; Gerlich, 2025). An in-depth investigation into how AIL influences university students’ IB is therefore both a theoretical imperative and a practical necessity for optimizing educational practices.

Existing research has generally confirmed that AIL plays a pivotal role in stimulating individuals’ IB (Chen and Xiao, 2025; Chun et al., 2025; Ji et al., 2025; Li H. et al., 2026a). At the cognitive level, AIL enhances IB by promoting flexible cognitive shifts and the restructuring of knowledge frameworks (Chen and Xiao, 2025; Xiang et al., 2025b). At the affective level, it helps alleviate the anxiety associated with interacting with AI technologies, thereby freeing psychological resources that would otherwise be consumed by negative emotions and creating space for creative activities (Hwang and Wu, 2025; Xiang et al., 2025b). Moreover, this effect does not occur in isolation but is jointly moderated by individual psychological traits and environmental and social factors (Weng, 2025; Li H. et al., 2026a). Although indirect evidence suggests that AIL may positively influence IB through enhanced cognitive flexibility (CF) and mitigated AI anxiety (AIA), systematic research integrating these variables into a unified framework remains scarce (Cengiz and Peker, 2025; Chen and Xiao, 2025). To our knowledge, no prior study has incorporated AIL, AIA, CF, and IB within a single analytical model to examine their interplay. At the same time, AIL is inherently multidimensional, comprising knowledge, skills, values, and ethical awareness (Zhou et al., 2024). Due to differences in personal experiences, students may display diverse configurations across these dimensions, and aggregate-level, variable-centered evaluations risk masking this unobserved heterogeneity (Zhou et al., 2024; Zhang and Kang, 2026). This gap not only hinders the development of personalized interventions but may also lead to the misallocation or underuse of educational resources.

To address these challenges, the present study introduces affordance theory to construct a serial mediation model, aiming to elucidate how AIL influences university students’ IB through the mediating roles of AIA and CF. Simultaneously, a latent profile analysis (LPA) is employed to identify the latent classes of university students’ AIL, thereby revealing the heterogeneous structure within the population. This research design not only contributes to a more profound understanding of the psychological mechanisms through which AIL affects IB but also provides a novel perspective for examining the diverse configurations of AIL and their relationship with IB through the lens of individual differences.

## 2 Literature review

### 2.1 AIL and IB

AIL refers to the capacity to understand, use, and evaluate AI technologies while maintaining a critical awareness of their social and ethical implications (Ji et al., 2025). As a multidimensional construct, AIL encompasses core elements such as knowledge, skills, ethics, and values (Zhou et al., 2024). With the rapid development of AI, AIL is no longer an exclusive competence of domain experts but has become an essential digital literacy for everyone (Walter, 2024). Like computational thinking, it requires students to move beyond being mere end-users and learn to leverage AI to solve real-world problems (Hu et al., 2025). Students lacking AI knowledge and skills often face significant constraints in innovation and problem-solving (Wu and Zhang, 2025). Fundamentally, the core value of AIL lies in stimulating individuals’ exploratory spirit, encouraging them to continuously reflect on how to apply AI technologies to address real-world challenges and thereby fostering enhanced innovative capabilities through sustained practice (Lin and Chen, 2024). This aligns with the core tenet of affordance theory, which posits that objects in the environment convey “what can be done” with them based on their inherent properties (Bornstein and Gibson, 1980). As Koffka (2013) vividly illustrated, “Each thing says what it is… a fruit says ‘Eat me’; water says ‘Drink me’; thunder says ‘Fear me’; and woman says ‘Love me’”. In essence, affordance denotes what an object tells an individual it can be used for. From this perspective, the primary function of AIL is to deepen and broaden individuals’ perception of AI affordances—and only those affordances genuinely perceived can substantially influence behavior (Volkoff and Strong, 2013; Lu et al., 2025). This theoretical expectation is supported by Tripon (2025) study. Tripon (2025) argued that the core function of information literacy is to help individuals critically transform raw information and data into actionable knowledge, a process that promotes deeper knowledge construction and innovative application, thereby enhancing IB. This perspective has been empirically extended to AIL (Weikang and Chenghua, 2026). Research shows that students with higher AIL are better able to perceive the practical value of AI tools such as ChatGPT, DeepSeek, and Gemini, and are more adept at using them for personalized learning and independent thinking, converting AI-generated information into actionable innovation resources and thus improving their innovative ability (Zhou and Peng, 2025). Further studies indicate that integrating AIL into educational practice can effectively cultivate students’ innovative capacity (Tripon, 2025; Wu and Zhang, 2025). Specifically, by deepening students’ perception of AI affordances, AIL education guides them to reflect during tool exploration, strengthens their ability to evaluate and utilize AI-generated information, and fosters an awareness of information as a callable resource and creative material, thereby facilitating IB (Tripon, 2025; Wu and Zhang, 2025). Collectively, these findings point to a fundamental trend: as students’ AIL improves, their creative capabilities correspondingly increase (Lu et al., 2025).

Therefore, this study proposes the following hypothesis 1 (H1): AIL is positively associated with university students’ IB.

### 2.2 The mediating role of AIA

AIA is defined as the apprehension or fear individuals experience when anticipating the negative consequences and risks associated with AI applications (Li and Huang, 2020). Such anxiety not only hinders students’ rational use of AI technologies but also raises concerns about their future learning and employment prospects, adversely affecting their holistic development (Ge et al., 2025). Notably, AIA is closely related to individuals’ attitudes toward AI (Kaya et al., 2024). Hopcan et al. (2024) found that those who recognize the significance and impact of AI tend to exhibit lower levels of fear toward it. Concurrently, university students’ attitudes toward AI are positively correlated with their AIL, and both are negatively correlated with AIA (Cengiz and Peker, 2025). Students with greater AI knowledge and familiarity with relevant principles and terminology are more likely to perceive the positive impacts of AI on personal and societal development, and as a result, they hold more favorable views of AI and correspondingly lower levels of AIA (Al Saad et al., 2022). Furthermore, alleviating AIA releases psychological resources that facilitate the development of IB (Fan et al., 2025). Research has shown that different levels of anxiety exert differential effects on individuals’ IB (Xiang et al., 2025b, 2025a). Specifically, individuals with high anxiety exhibit a significant decline in both creative activities and achievements (Xiang et al., 2025a). When individuals experience anxiety toward AI, such negative emotions may erode their confidence in their own innovative capabilities—they may worry that their skills will be replaced by AI, or that their innovative efforts hold little value in the AI era, leading them to avoid challenging innovative tasks (Öztırak, 2023).

Accordingly, this study proposes the following hypothesis (H2): AIA significantly mediates the relationship between AIL and university students’ IB.

### 2.3 The mediating role of CF

CF is defined as an individual’s awareness that multiple options exist in any given situation, coupled with their willingness to adapt flexibly and their self-efficacy in doing so (Martin and Rubin, 1995). Research indicates that when university students’ AIL is low, the mediating role of CF on IB disappears, suggesting a potentially important link between AIL and CF (Chen and Xiao, 2025; Karaoglan Yilmaz et al., 2025). The widespread use of AI has profoundly changed how students access information (Von Garrel and Mayer, 2023). AI-generated or AI-recommended content often defies traditional interpretation, with its diversity and complexity far exceeding that of conventional learning materials (Guingrich and Graziano, 2024). This requires individuals to proactively identify and evaluate information that can expand their cognitive boundaries while adjusting their thinking to specific contexts (Sebastian, 2025). In other words, only when individuals recognize the value of novel information are they likely to recalibrate their cognitive schemas, reconstruct their understanding of existing knowledge, and thereby become more adept at navigating complex problems flexibly (Ng et al., 2024). The ability to effectively use AI to solve real-world problems, therefore, constitutes effective cognitive training for CF (Sarna, 2025). Accordingly, enhancing AIL should help students better identify and use information resources conducive to cognitive reconstruction, thereby strengthening their CF (Chen and Xiao, 2025). CF is also widely recognized as a key determinant of IB (Xu et al., 2019). According to the dual pathway to creativity model, one route to IB involves achieving creative outcomes through flexible information processing (Nijstad et al., 2010; Baas et al., 2013). Empirical evidence demonstrates that individuals with elevated CF exhibit significantly more pronounced IB (Baas et al., 2013). When individuals can approach problems from multiple conceptual categories and perspectives, their thinking becomes more flexible—for example, recognizing that a brick can serve not only as a construction material but also as a counterweight or a weapon (Baas et al., 2013). Such cognitive flexibility fosters the generation of numerous creative ideas and solutions by bridging seemingly unrelated knowledge domains, thereby producing unexpected creative outcomes (Paulus and Brown, 2007).

Based on the above reasoning, this study proposes the following hypothesis (H3): CF significantly mediates the relationship between AIL and university students’ IB.

### 2.4 The serial mediating role of AIA and CF

The rapid advancement of AI technologies since the beginning of the 21st century has profoundly reshaped human learning and lifestyles (Von Garrel and Mayer, 2023). Individuals who struggle to adapt to these transformations may fall into emotional distress marked by anxiety and fear(İnci Kuzu, 2025). Specifically, university students experiencing AIA often display marked hesitation, avoidance, and even resistance when using AI tools (Taş and Turanlıgil, 2020). Such persistent negative usage patterns not only impair effective interaction with emerging technologies but also inhibit the development of CF, providing solid empirical and theoretical support for AIA as an antecedent of reduced CF (Kaya and Keskin, 2026). Further research has identified multiple mechanisms underlying this inhibitory effect. On the one hand, avoidance of and resistance toward AI reduce individuals’ exposure to diverse information and novel problem-solving situations, which over time can homogenize cognitive experience and rigidify thinking pathways, making cross-context transfer and flexible strategy adjustment increasingly difficult (Qi et al., 2013; Xiang et al., 2025b). On the other hand, anxiety itself consumes limited cognitive resources, impairing executive control, responsiveness, and task-switching capacity, thereby further constraining CF development (Mashayekh, 2025; Xiang et al., 2025b). Notably, AIL plays an important moderating role throughout these psychological processes (Chen and Xiao, 2025; Karaoglan Yilmaz et al., 2025). Specifically, enhanced AIL enables students to develop a more critical and objective understanding of AI, reducing their perception of AI as a threat driven by uncertainty and effectively alleviating AIA (Chen and Xiao, 2025). As AIA diminishes, students can free cognitive resources for deeper thinking and more flexible shifting; simultaneously, their avoidance behaviors decrease, and they become willing to actively explore new functions and diverse usage patterns (Vytal et al., 2013; Karaoglan Yilmaz et al., 2025). This exploratory process exposes them to more varied information and problem contexts, which in turn continuously exercises and strengthens their CF (Lee et al., 2024). Students with higher CF are then more inclined to break away from fixed mindsets, skillfully establishing novel connections among seemingly unrelated pieces of information and deriving creative insights, solution strategies, or new ideas, ultimately driving the emergence of IB (Hu and Wang, 2023; Herault et al., 2024).

**Figure 1:**
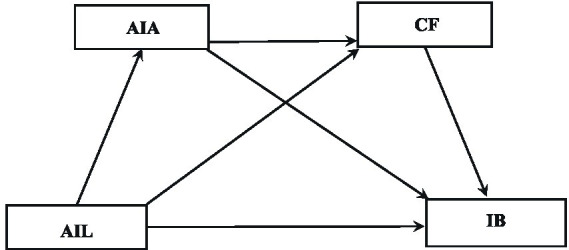
The proposed mediation model.

Accordingly, this study proposes the following hypothesis (H4): AIA and CF exert a significant serial mediating effect on the relationship between AIL and university students’ IB (see Figure 1).

### 2.5 Latent structural differences in AIL

University students’ AIL is not a homogeneous construct; rather, it manifests pronounced heterogeneity that cannot be adequately captured by a single continuous dimension (Li X. et al., 2026b). Thus, it is more rigorous and appropriate to conceptualize AIL as distinct latent subtypes rather than as a uniform continuous variable (Zhu et al., 2026). Extant literature has yielded valuable preliminary explorations in this regard (Zhang and Kang, 2026; Zhu et al., 2026). For instance, using LPA, Zhang and Kang (2026) identified four typical AIL subtypes among university students, while Zhu et al. (2026) categorized students into high, moderate, and low tiers based on their literacy proficiency. Notably, the heterogeneity inherent in AIL significantly impacts university students’ IB, underscoring the importance of examining the structural configurations of AIL to better understand its nexus with IB (Li X. et al., 2026b; Wang et al., 2026). As a multidimensional competency encompassing knowledge, skills, values, and ethical awareness, AIL is indispensable for students’ sustainable development in the AI era (Ji et al., 2025). Driven by differences in individual developmental trajectories, educational backgrounds, and psychological traits, students may display highly diversified configurational patterns across these dimensions (Li X. et al., 2026b; Wang et al., 2026). Ecological systems theory offers a robust lens for explaining this phenomenon, positing that individual development is shaped by nested environmental systems, particularly at the microsystem level, which includes family, school, and peers (Bronfenbrenner, 1979). Within these dynamic systems, university students gradually crystallize distinct AIL subtypes shaped by their contextualized, developmentally relevant experiences (Bronfenbrenner, 1979; Zhu et al., 2026). Identifying these unobserved latent subpopulations yields substantial theoretical and practical implications (Medina-Gual and Parejo, 2025; Wang et al., 2026). On the one hand, it facilitates the unmasking of intra-group heterogeneity, thereby deepening our comprehension of the structural configurations of AIL and their underlying formative mechanisms. On the other hand, it equips educators with actionable theoretical frameworks for implementing precision pedagogy and targeted personalized interventions, which in turn offers critical insights for fostering university students’ IB.

Accordingly, this study proposes the following hypothesis (H5): The pathways through which AIL influences university students’ IB via AIA and CF differ significantly across distinct latent profiles.

## 3 Methods

### 3.1 Participants

**Figure 2:**
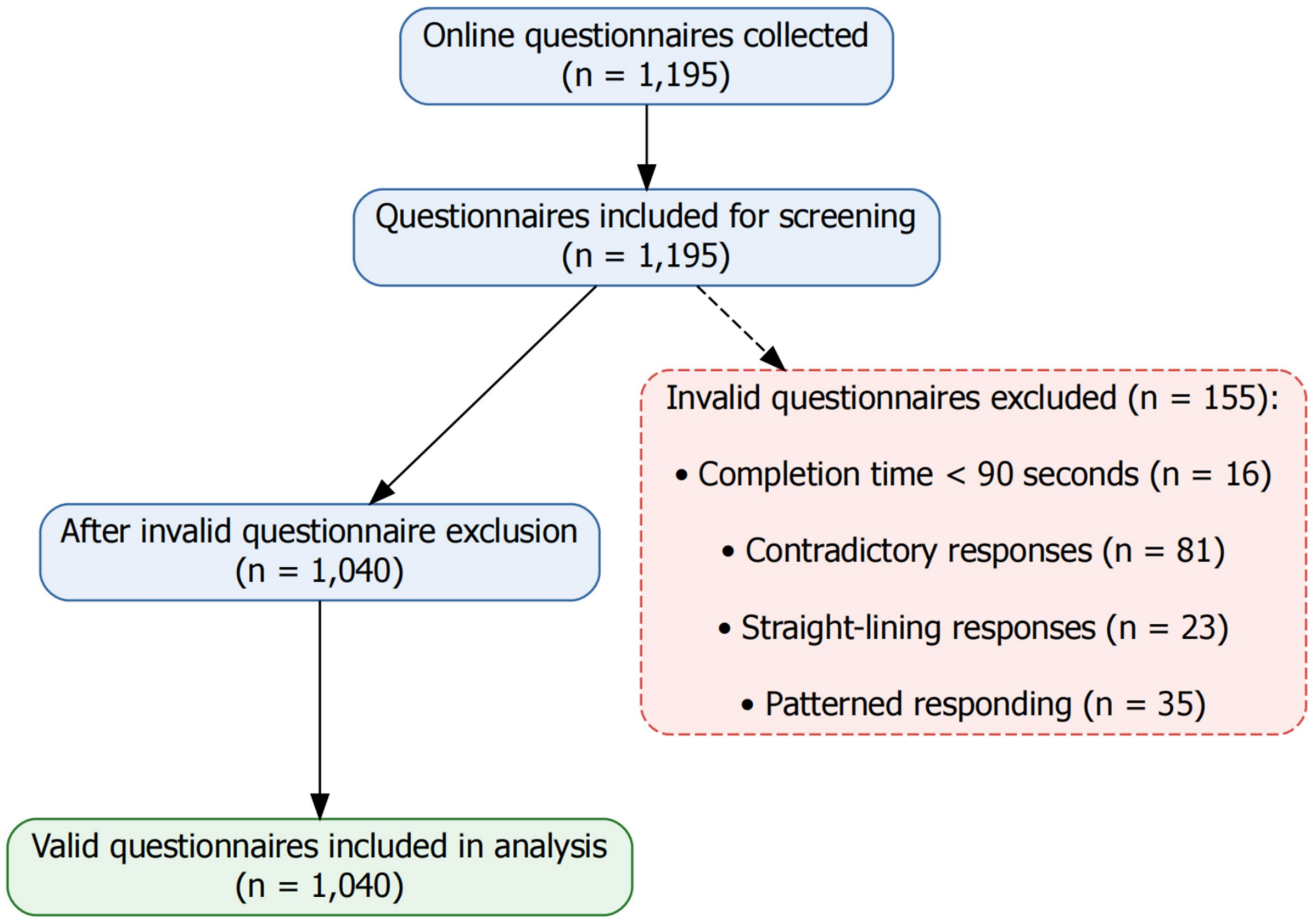
Steps in the screening process for research samples.

A convenience sampling method was utilized. Participants were recruited via the online survey platform Sojump,^1^ and the sample consisted of university students from various provinces in China. Prior to participation, all respondents were fully briefed on the research objectives, the strict anonymity of their responses, and their right to withdraw from the study at any time without penalty. Electronic informed consent was obtained from each participant. The study protocol was reviewed and approved by the Biomedical Research Ethics Committee of Henan University. A total of 1,195 online questionnaires were distributed and retrieved. During the data cleaning process, invalid responses were excluded based on the following predetermined criteria: unrealistically short completion time (<90 s, *n* = 16), contradictory responses (*n* = 81), straight-lining or invariant responses across all items (*n* = 23), and recognizable patterned responding (*n* = 35). Following the application of these exclusion criteria, a final sample of 1,040 valid questionnaires was retained for subsequent analyses, yielding an effective response rate of 87.0% (see Figure 2). The final analytical sample comprised 549 males (52.79%) and 491 females (47.21%), with a mean age of 21.80 years (*SD* = 2.03), and all participants were at least 18 years old (see Table 1). Regarding academic standing, the distribution was as follows: 142 freshmen (13.65%), 140 sophomores (13.46%), 659 juniors (63.37%), and 99 seniors (9.52%).

**Table 1:** Distribution of basic information (*n* = 1,040).

Demographic variables		Number	Proportion%
Age (*M ± SD*)	21.80 ± 2.03	1,040	100%
Gender	Male	549	52.79%
	Female	491	47.21%
Grade	Freshmen	142	13.65%
	sophomores	140	13.46%
	Juniors	659	63.37%
	Seniors	99	9.52%

M, mean; SD, standard deviation.

### 3.2 Measurement instruments

*AIL Scale*. The AIL scale, adapted from Zhou et al. (2024), consists of 25 items across four dimensions: knowledge, skills, values, and ethical awareness. Items were rated on a 7-point Likert scale ranging from 1 (strongly disagree) to 7 (strongly agree), with higher scores indicating higher levels of AIL. In this study, confirmatory factor analysis (CFA) indicated a good model fit, with the following indices: χ^2^/df = 2.54, RMSEA = 0.04, GFI = 0.95, NFI = 0.97, CFI = 0.98. The Cronbach’s *α* coefficient for the scale was 0.92. Additional details are provided in Supplementary Tables S1, S2.

*IB Scale*. IB was measured using the scale adapted from Liu and Shi (2009), which comprises five items as a single-dimension measure. Items were rated on a 7-point Likert scale, with higher scores reflecting higher levels of innovative behavior. CFA results showed a good model fit: χ^2^/df = 1.82, RMSEA = 0.03, GFI = 1.00, NFI = 1.00, CFI = 1.00. The Cronbach’s α coefficient for the scale was 0.83. Further details are presented in Supplementary Table S6.

*AIA Scale*. AIA was assessed using the scale developed by Wang and Wang (2022), which has been widely used in China (Chen et al., 2025; Liu et al., 2025). The scale consists of 21 items across four dimensions: learning anxiety, AI configuration anxiety, job replacement anxiety, and socio-technical blindness. Items were rated on a 7-point Likert scale, with higher scores indicating higher levels of AI anxiety. CFA indicated a good model fit: χ^2^/df = 1.73, RMSEA = 0.03, GFI = 1.00, NFI = 0.99, CFI = 1.00. The Cronbach’s α coefficient for the scale was 0.91. Supplementary information is provided in Supplementary Tables S3, S4.

*CF Scale*. CF was measured using the Chinese version of the CF Scale adapted by Qi et al. (2013). The scale comprises 13 items (including four reverse-coded items) as a single-dimension measure, rated on a 6-point Likert scale, with higher scores indicating greater cognitive flexibility. CFA results showed a good model fit: χ^2^/df = 1.03, RMSEA = 0.10, GFI = 1.00, NFI = 0.99, CFI = 1.00. The Cronbach’s α coefficient for the scale was 0.81. Supplementary Table S5 provides further details.

### 3.3 Statistical analysis

Data processing and statistical analysis were performed using Microsoft Excel, SPSSAU, and Mplus 8.3. First, Microsoft Excel was used to preprocess the raw data. Through logical consistency checks, questionnaires with patterned responses or excessively short completion times were excluded to ensure data integrity and reliability. Subsequently, the screened valid data were imported into the SPSSAU online statistical platform for the following analyses. First, reliability and validity of each scale were assessed using indicators such as χ^2^/df, RMSEA and Cronbach’s α. Second, descriptive statistics of the main study variables were calculated, and Pearson correlation analysis was conducted to examine the relationships among the variables. Third, Harman’s single-factor test was used to assess common method bias, evaluating whether the total variance explained by a single factor fell within an acceptable range. Finally, a chain mediation model was constructed using the Bootstrap resampling method (5,000 resamples, 95% confidence interval [CI]) to examine the mediating effects and path relationships of AIA and CF in the relationship between AIL and IB. To further uncover the heterogeneous structure of university students’ AIL, LPA was conducted using Mplus 8.3, with the 25 items of the AI literacy scale as continuous indicators. This person-centered approach classifies individuals with similar behavioral characteristics into the same category based on statistical models, thereby identifying potential subtypes within the population. Model fit was evaluated using multiple indices, including the Akaike Information Criterion (AIC), Bayesian Information Criterion (BIC), sample-size adjusted BIC (aBIC), entropy, Lo–Mendell–Rubin likelihood ratio test (LMR), and Bootstrap likelihood ratio test (BLRT). Models with one to four profiles were fitted, and the optimal classification was determined based on statistical fit and theoretical interpretability. After identifying the latent profile structure, multi-group chain mediation analysis was performed. Specifically, the latent profiles derived from Mplus 8.3 were used as grouping variables, and the Bootstrap resampling method (5,000 resamples, 95%CI) was applied in SPSSAU to test for significant differences in the chain mediation paths across different groups.

Prior to the main analyses, the distribution of the age variable was examined. Although the Kolmogorov–Smirnov and Shapiro–Wilk tests were significant (*p* < 0.001), the skewness (0.772) and kurtosis (0.470) were within the acceptable range for approximate normality (skewness < |2|, kurtosis < |7|) (Hair et al., 2010). Thus, age was considered sufficiently close to normal for parametric analyses.

## 4 Results

### 4.1 Common method Bias test

Exploratory factor analysis revealed four factors with eigenvalues greater than 1. The first factor accounted for 19.893% of the total variance, which is below the critical threshold of 40%, indicating that common method bias was not a serious concern. In addition, we further examined common method bias for the AIA and AIL scales using confirmatory factor analysis, and the results likewise indicated no serious common method bias (see Supplementary Table S7 for details).

### 4.2 Descriptive statistics and correlation analysis

Descriptive statistics and correlation analyses were conducted for AIL, IB, AIA, and CF. As shown in Table 2, significant correlations were observed among the four variables (all *p* < 0.001), providing a prerequisite for testing the research hypotheses. Specifically, AIL was significantly positively correlated with IB (*r* = 0.329, *p* < 0.001) and CF (*r* = 0.346, *p* < 0.001), and significantly negatively correlated with AIA (*r* = −0.319, *p* < 0.001). IB was significantly positively correlated with CF (*r* = 0.283, *p* < 0.001) and significantly negatively correlated with AIA (*r* = −0.258, *p* < 0.001). AIA was significantly negatively correlated with CF (*r* = −0.296, *p* < 0.001).

**Table 2:** Mean, standard deviation, and correlations between the variables.

Variable	M	SD	1	2	3	4
1. AIL	5.094	0.988	1			
2. IB	5.296	1.280	0.329***	1		
3. AIA	3.713	1.105	−0.319***	−0.258***	1	
4. CF	4.378	0.953	0.346***	0.283***	−0.296***	1

*p < 0.05, **p < 0.01, ***p < 0.001.

### 4.3 Serial mediation analysis

As shown in Table 3, AIL was significantly and positively associated with IB (*β* = 0.226, *p* < 0.001), and CF also showed a significant positive association with IB (*β* = 0.166, *p* < 0.001). In contrast, AIA was significantly and negatively associated with IB (*β* = −0.137, *p* < 0.001). Furthermore, AIL was positively associated with CF (*β* = 0.280, *p* < 0.001), while AIA was negatively associated with CF (*β* = −0.206, *p* < 0.001). Additionally, AIL showed a significant negative association with AIA (*β* = −0.320, *p* < 0.001). These results are consistent with the hypothesized associations, thereby supporting H1.

**Table 3:** Hierarchical multiple linear regression analysis results.

Variables	AIA		CF		IB	
	*β*	*t*	*β*	*t*	*β*	*t*
Gender	0.045	1.515	−0.018	−0.640	0.004	0.124
Age	0.007	0.226	0.008	0.263	0.022	0.753
Grade	−0.029	−0.985	−0.002	−0.069	−0.001	−0.052
AIL	−0.320	−10.861***	0.280	9.317***	0.226	7.208***
AIA			−0.206	−6.829***	−0.137	−4.431***
CF					0.166	5.321***
*R^2^*	0.105		0.162		0.158	
*∆R^2^*	0.100		0.157		0.153	
*F*	27.171***		33.339***		27.754***	

*p < 0.05, **p < 0.01, ***p < 0.001.

**Figure 3:**
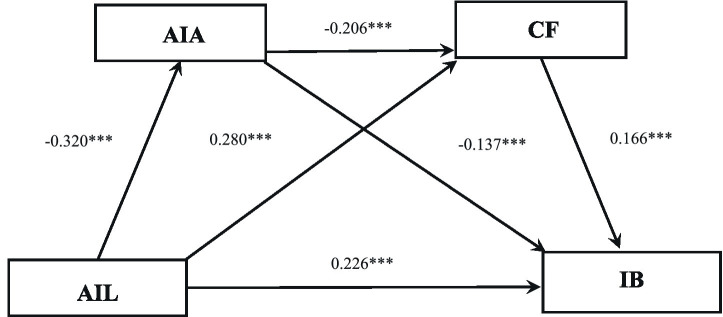
Chain mediation effects of AIA and CF in the relationship between AIL and IB.

Table 4 and Figure 3 indicate that among the three mediation pathways: (1) In Path 1, namely the AIL → AIA → IB pathway, the indirect effect of AIA was 0.057, 95%CI [0.030, 0.086], accounting for 13.44% of the total effect. (2) In Path 2, namely the AIL → CF → IB pathway, the indirect effect of CF was 0.060, 95%CI [0.035, 0.090], accounting for 14.15% of the total effect. (3) In Path 3, namely the AIL → AIA → CF → IB pathway, the serial indirect effect of AIA and CF was 0.014, 95%CI [0.008, 0.022], accounting for 3.30% of the total effect. Consequently, H 2, 3, and 4 were supported.

**Table 4:** Direct and indirect effects in the multiple mediator model.

Model	Effect	Boot SE	Boot LLCI	Boot ULCI	Relative mediation effect
Total effect	0.424	0.038	0.350	0.499	100%
Total direct effect	0.293	0.041	0.213	0.373	69.10%
Total indirect effect	0.131	0.020	0.093	0.172	38.90%
AIL⇒AIA ⇒ IB	0.057	0.014	0.030	0.086	13.44%
AIL⇒CF ⇒ IB	0.060	0.014	0.035	0.090	14.15%
AIL⇒AIA ⇒ CF ⇒ IB	0.014	0.004	0.008	0.022	3.30%

Boot SE, Standard error of indirect effects; Boot LLCI, the lower bound of the 95% confidence interval; Boot ULCI, the upper limit of the 95% confidence interval (Percentile Bootstrap Method with Bias Correction).

### 4.4 Latent profile analysis of AIL

In this study, a series of latent profile models, ranging from one to four classes, were estimated to identify the latent profiles of AIL. The model fit indices for these four models are summarized in Table 5. Regarding the fit indices for latent profile analysis, lower values of the AIC, BIC, and aBIC indicate superior model fit. An Entropy value exceeding 0.8 signifies a classification accuracy of over 90%, with values closer to 1 indicating optimal classification precision (Wang et al., 2017). Furthermore, significant *p*-values (*p* < 0.05) for the LMR and BLRT suggest that a k-class model provides a significantly better fit than a (k-1)-class model. Across the four specified models, the values of AIC, BIC, and aBIC consistently decreased as the number of latent classes increased. Additionally, the Entropy values for all four models exceeded 0.8. The LMR and BLRT *p*-values for the first three profile models reached statistical significance (*p* < 0.05). However, for the 4-class model, the LMR test yielded a non-significant value (*p* = 0.30, *p* > 0.05). Weighing the optimal fit indices simultaneously against the parsimony of the model and the theoretical interpretability of the results (Rindskopf, 2003), the 3-class model was ultimately determined to be the most optimal and well-fitting model for this study.

**Table 5:** Model fit indicators for different Profiles of AIL.

Class	AIC	BIC	aBIC	Entropy	LMR (*P*)	BLRT (*P*)	Group size	Class probability
1	101287.387	101534.736	101375.930	—	—	—	1,040	—
2	95969.266	96345.236	96103.850	0.918	<0.001	<0.001	358/682	0.34/0.66
3	**94675.685**	**95180.277**	**94856.311**	**0.894**	<0.001	<0.001	**159/385/496**	**0.15/0.37/0.48**
4	94315.276	94948.489	94541.944	0.836	0.30	<0.001	115/260/399/266	0.11/0.25/0.38/0.26

### 4.5 Characteristics of AIL latent profiles

**Figure 4:**
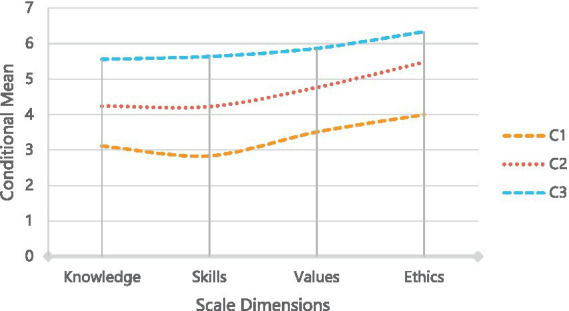
Estimated conditional means of latent classes for AIL.

The latent profiles of AIL are illustrated in Figure 4. Class 1 (C1), comprising 159 students (15.29%), exhibited the lowest estimated scores across all dimensions and was thus labeled the “Low AIL” profile. Class 2 (C2), consisting of 385 students (37.02%), displayed intermediate scores—higher than those of C1 but lower than those of C3—and was designated the “Moderate AIL” profile. Class 3 (C3), encompassing the largest proportion of the sample with 496 students (47.69%), consistently scored the highest across all dimensions and was accordingly termed the “High AIL” profile. As presented in Table 6, significant differences were observed in the mean scores on the AIL scale among the three distinct latent profiles of university students.

**Table 6:** Descriptive statistics and difference tests of AIL among the three groups (*M ± SD*).

Dimension	Category			*F*	*Post Hoc* Test
	C1 (*n* = 159)	C2 (*n* = 385)	C3 (*n* = 496)		
Ethics	3.990 ± 0.807	5.461 ± 0.540	6.343 ± 0.437	1156.822***	C3 > C2 > C1
Values	3.494 ± 0.848	4.759 ± 0.692	5.866 ± 0.619	793.735***	C3 > C2 > C1
Skills	2.828 ± 0.645	4.208 ± 0.654	5.645 ± 0.589	1411.587***	C3 > C2 > C1
Knowledge	3.099 ± 1.005	4.232 ± 1.137	5.562 ± 0.903	421.177***	C3 > C2 > C1

*p < 0.05, **p < 0.01, ***p < 0.001.

### 4.6 The relationship between AIL latent profiles and IB

To explore the relationship between AIL and university students’ IB, a one-way analysis of variance (ANOVA) was conducted, utilizing the latent profiles of AIL as the independent variable and the mean scores of IB as the dependent variable (see Table 7). The results indicated significant differences in IB among university students across the distinct latent profiles of AIL (*F* = 51.045, *p* < 0.001). Furthermore, *post hoc* comparisons revealed that the mean scores of IB across the latent profiles followed a descending order of C3 > C2 > C1.

**Table 7:** Descriptive statistics and difference tests of IB among the three groups (*M ± SD*).

Variable	Category			*F*	*Post Hoc* Test
	C1 (*n* = 159)	C2 (*n* = 385)	C3 (*n* = 496)		
IB	4.652 ± 1.403	5.082 ± 1.331	5.669 ± 1.062	51.045***	C3 > C2 > C1

*p < 0.05, **p < 0.01, ***p < 0.001.

### 4.7 Chain mediation analysis based on AIL latent profile categories

To investigate whether the mediating roles of AIA and CF in the relationship between AIL and IB are moderated by the latent profile categories of AIL among college students, a multi-group chain mediation model was employed. Specifically, a chain mediation analysis was conducted using the three latent profiles as the grouping variable, with Group C2 serving as the reference group. The results (see Table 8) indicated that, compared to Group C2, Group C1 was significantly and negatively associated with CF (*β* = −0.071, *p* < 0.05) and IB (*β* = −0.081, *p* < 0.05), but significantly and positively associated with AIA (*β* = 0.147, *p* < 0.001). Conversely, relative to Group C2, Group C3 exhibited significant positive associations with CF (*β* = 0.245, *p* < 0.05) and IB (*β* = 0.149, *p* < 0.05), and a significant negative association with AIA (*β* = −0.194, *p* < 0.001). Furthermore, AIA was negatively associated with both CF (*β* = −0.217, *p* < 0.001) and IB (*β* = −0.151, *p* < 0.001), while CF was positively associated with IB across the groups (*β* = 0.174, *p* < 0.001).

**Table 8:** Hierarchical multiple linear regression analysis results (Vs. C2).

Variables	AIA		CF		IB	
	*β*	*t*	*β*	*t*	*β*	*t*
Gender	0.047	1.575	−0.021	−0.725	0.003	0.091
Age	0.007	0.234	0.008	0.275	0.022	0.759
Grade	−0.028	−0.919	−0.004	−0.150	−0.003	−0.117
C3	−0.194	−5.947***	0.245	7.732***	0.149	4.530***
C1	0.147	4.499***	−0.071	−2.243*	−0.081	−2.536*
AIA			−0.217	−7.307***	−0.151	−4.902***
CF					0.174	5.518***
*R^2^*	0.084		0.165		0.149	
*∆R^2^*	0.079		0.159		0.142	
*F*	15.889***		29.051***		22.498***	

*p < 0.05, **p < 0.01, ***p < 0.001.

**Figure 5:**
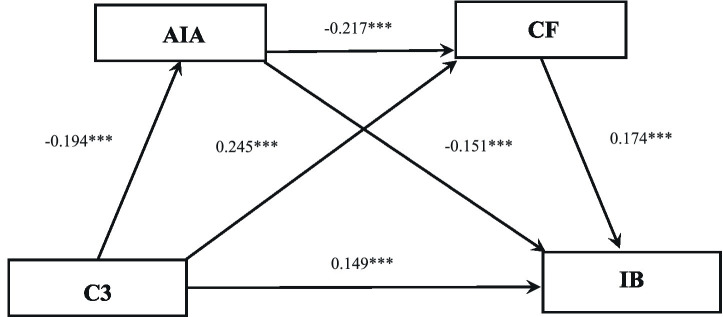
Chain mediation effects of AIA and CF in the relationship between AIL and IB (C3 vs. C2).

Table 9 and Figure 5 illustrate the mediation effect analysis for Group C3 relative to Group C2. The results revealed the following across the three mediating pathways: (1) In Path 1 (AIL → AIA → IB), the indirect effect of AIA was 0.075 (95%CI [0.038, 0.121]), accounting for 12.84% of the total effect; (2) In Path 2 (AIL → CF → IB), the indirect effect of CF was 0.109 (95%CI [0.063, 0.162]), accounting for 18.66% of the total effect; and (3) In Path 3 (AIL → AIA → CF → IB), the sequential indirect effect of AIA and CF was 0.019 (95%CI [0.010, 0.030]), accounting for 3.25% of the total effect.

**Table 9:** Direct and indirect effects in the multiple mediator model (C3 Vs. C2).

Model	Effect	Boot SE	Boot LLCI	Boot ULCI	Relative mediation effect
Total effect	0.584	0.083	0.421	0.748	100%
Total direct effect	0.382	0.084	0.216	0.547	65.41%
Total indirect effect	0.203	0.034	0.138	0.271	34.76%
AIL⇒AIA ⇒ IB	0.075	0.021	0.038	0.121	12.84%
AIL⇒CF ⇒ IB	0.109	0.025	0.063	0.162	18.66%
AIL⇒AIA ⇒ CF ⇒ IB	0.019	0.005	0.010	0.030	3.25%

**Figure 6:**
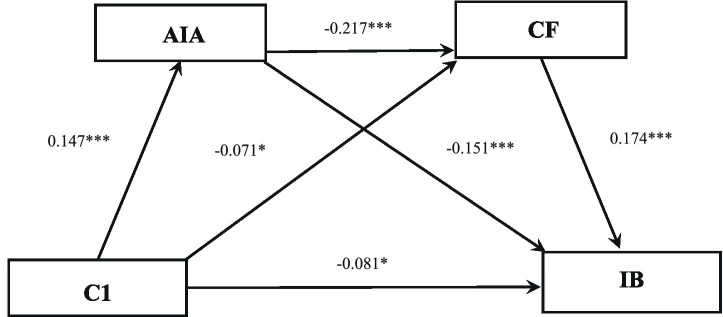
Chain mediation effects of AIA and CF in the relationship between AIL and IB (C1 vs. C2).

Table 10 and Figure 6 present the mediation effect analysis for Group C1 relative to Group C2. The findings indicated the following across the three mediating pathways: (1) In Path 1 (AIL → AIA → IB), the indirect effect of AIA was −0.079 (95%CI [−0.133, −0.035]), representing 18.42% of the total effect; (2) In Path 2 (AIL → CF → IB), the indirect effect of CF was −0.044 (95%CI [−0.095, −0.002]), representing 10.26% of the total effect; and (3) In Path 3 (AIL → AIA → CF → IB), the sequential indirect effect of AIA and CF was −0.020 (95%CI [−0.034, −0.009]), representing 4.66% of the total effect. Consequently, H5 was supported.

**Table 10:** Direct and indirect effects in the multiple mediator model (C1 vs. C2).

Model	Effect	Boot SE	Boot LLCI	Boot ULCI	Relative mediation effect
Total effect	−0.429	0.116	−0.656	−0.203	100%
Total direct effect	−0.287	0.113	−0.510	−0.065	66.90%
Total indirect effect	−0.142	0.037	−0.218	−0.075	33.10%
AIL⇒AIA ⇒ IB	−0.079	0.025	−0.133	−0.035	18.42%
AIL⇒CF ⇒ IB	−0.044	0.024	−0.095	−0.002	10.26%
AIL⇒AIA ⇒ CF ⇒ IB	−0.020	0.006	−0.034	−0.009	4.66%

## 5 Discussion

This study examined the relationship between AIL and IB and its underlying mechanisms. The results showed a significant positive association between AIL and IB. After incorporating AIA and CF as mediators, both the direct and indirect associations of AIL with IB remained significant, suggesting that AIL relates to IB not only directly but also through the independent and sequential mediating pathways of AIA and CF. It is worth noting that the chain mediating effect of AIA and CF accounts for only 3.30% of the total effect; therefore, caution should be exercised when interpreting this specific finding. Additionally, LPA was employed to identify the latent categories of college students’ AIL, classifying them into three distinct profiles: low AIL (C1), moderate AIL (C2), and high AIL (C3). Further analysis revealed significant differences in the impact of these latent profiles on students’ IB, a variance that was consistently reflected in the profile-based chain mediation analysis.

### 5.1 The direct effect of AIL on IB

The present study found that AIL remained significantly associated with IB even after accounting for the mediating variables, supporting H1. This finding is broadly consistent with existing literature (Lu et al., 2025; Polat, 2025; Weikang and Chenghua, 2026; Zhang, 2026). While AI brings unprecedented efficiency and convenience to university students’ academic and daily lives, it also carries latent risks such as AIA, technological over-reliance, and cognitive inertia (Zhang et al., 2023). Cultivating AIL has thus become essential to ensure that students maintain autonomous innovative awareness and behavior rather than being supplanted by technology in the AI era. Research indicates that the inherently critical nature of AIL stimulates students’ IB, helping them preserve the capacity for independent thinking, which in turn enhances their innovative performance (Zhang, 2026). The four dimensions of AIL—knowledge, skills, values, and ethical awareness—each positively contribute to IB (Weikang and Chenghua, 2026). The enhancement of knowledge and skills provides the technical and operational foundation for various tasks, strengthening students’ capacities for information processing, innovation generation, and practical application. Ethical awareness, meanwhile, ensures the sustainability and reliability of the innovation process by promoting fairness, circumventing biases, and removing ethical barriers. The capacity for value judgment regarding AI further stimulates subjective agency and fosters positive attitudes, thereby propelling the generation of value-oriented innovative concepts and the sustained development of IB (Floridi et al., 2018). Drawing on affordance theory, elevated AIL also enables a deeper understanding of AI’s advantages and limitations—particularly in functionality, interactivity, and convenience—lowering the threshold for AI utilization and catalyzing IB (Wang et al., 2024; Lu et al., 2025). Our findings diverge, however, from certain studies (Ji et al., 2025). This discrepancy may stem from the model proposed by Ji et al., in which AIL influences IB through the mediating roles of psychological need satisfaction, creative self-efficacy, and self-regulated learning (Ji et al., 2025). These three variables together construct a strong explanatory pathway that may fully mediate the direct effect. In contrast, the present study includes AIA and CF as mediators, both of which exert only partial mediating effects, thus preserving the direct association between AIL and IB. Despite these methodological differences, the core perspective of Ji et al. remains highly consistent with ours: as an emerging competency for human–AI collaboration, AIL enhances the efficiency and quality of individuals’ innovative activities carried out with technological support (Ji et al., 2025).

### 5.2 The mediating role of AIA

The present study further found that AIL was indirectly associated with university students’ IB through the mediating role of AIA, thereby supporting H2. This finding aligns with Newman’s et al. (2018) view that IB is inherently a dynamic self-regulatory process. In human–AI collaboration contexts, students with higher AIL are more receptive to emerging technologies and exhibit less fear and resistance toward AI (Mashayekh, 2025). Such a positive psychological state bolsters their innovative confidence, making them more capable of proposing and implementing creative solutions to real-world problems. Research by Weng (2025) supports this view, indicating that students who hold cautious attitudes toward AI are prone to developing apprehensions that subsequently constrain their IB. From the perspective of affordance theory, enhancing AIL fundamentally broadens students’ cognitive boundaries regarding AI and elevates their perceived affordances of the technology (Bornstein and Gibson, 1980; Lu et al., 2025). Specifically, students with superior AIL can astutely recognize AI’s profound functionalities in information integration, cross-disciplinary knowledge connection, and creative generation, and consequently perceive AI as a collaborator in the innovation process rather than a threat to their subjective agency (Chen and Xiao, 2025).

### 5.3 The mediating role of CF

Furthermore, the study found that AIL was indirectly associated with university students’ IB through the mediating role of CF, supporting H3. This finding echoes (Chen and Xiao, 2025) observation that CF significantly impacts IB only among students with high AIL. Delving into the underlying reasons, Karaoglan Yilmaz et al posit that CF is inextricably linked to an individual’s level of domain-specific knowledge mastery (Karaoglan Yilmaz et al., 2025). University students with higher AIL typically possess a more profound comprehension of AI-related knowledge and skills. This establishes a robust practical foundation for their flexible utilization of AI tools, enabling them to dynamically calibrate the AI tools employed in accordance with specific contextual demands, thereby effectively catalyzing the generation of IB. In addition, a substantial body of research shows that higher AIL is also associated with stronger critical thinking, a core component of CF. These students can grasp the ethical implications and inherent limitations of AI, critically examine its strengths and weaknesses, and thus avoid blind compliance or technological over-reliance (Hao et al., 2024; Walter, 2024). This cognitive paradigm, which fully activates subjective agency, helps transform AI from a mere tool into a collaborative partner, effectively stimulating and sustaining IB.

### 5.4 The serial mediating role of AIA and CF

This study further revealed that AIL’s relationship with IB also operated through the serial mediating role of AIA and CF, thereby supporting H4. This result lends further empirical support to affordance theory in the context of human-AI collaborative innovation (Koffka, 2013; Volkoff and Strong, 2013; Lu et al., 2025). Specifically, students with high AIL were able to effectively perceive the affordances of AI tools without seeing them as a threat, and their AIA levels were correspondingly lower. This low-anxiety state freed cognitive resources, enabling them to switch flexibly between perspectives and establish novel connections when engaging with diverse AI-generated information, thereby demonstrating higher CF (Lee et al., 2024; Karaoglan Yilmaz et al., 2025). This process not only enhanced their learning and work efficiency but also freed time and mental space for independent thinking, which in turn stimulated more novel ideas and IB (Cañas et al., 2003). It should be noted, however, that the serial mediation effect was relatively modest, accounting for only 3.30% of the total effect, and its practical significance should therefore be interpreted with caution. Even so, the existence of this pathway indicates that AIL’s influence on IB can be transmitted indirectly through the serial mediation of AIA and CF. This finding empirically extends the scope of affordance theory and, by revealing a key regulative mechanism through which AI facilitates IB, offers new clues for understanding the psychological processes underlying human-AI interaction.

### 5.5 Path differences across latent profiles of AIL

Furthermore, this study identified three distinct latent profiles of university students’ AIL, with significant path differences across these profiles, supporting H5. With C2 as the reference group, C3 performed better across all three indirect pathways, whereas C1 consistently performed worse. From a theoretical perspective, the differences among these three profiles reflect not only variation in dimensional scores but also three typical adaptation patterns in human-AI collaborative contexts. Low-AIL students tended to perceive AI as a threat to their employment and personal development, a perception linked to elevated AIA (Jiang and Chen, 2026). This anxiety was associated with avoidant or defensive attitudes toward AI, which reduced opportunities for active exploration and experimentation. Over time, such avoidance may lead to homogenized cognitive experiences and rigid thinking, ultimately constraining innovative thinking and ability (Zhu et al., 2026). On the other hand, insufficient AI education in some regions is a key factor contributing to low AIL levels among university students (Beckman et al., 2025). A prolonged lack of systematic AI learning and practice opportunities can make these students more susceptible to a cascade of challenges associated with low AIL, including heightened anxiety, cognitive rigidity, and constrained innovation (Satoto et al., 2025). Moderate-AIL students, by contrast, possessed a foundational understanding of AI and a generally positive attitude, and they exhibited relatively lower AIA (Cengiz and Peker, 2025). However, due to a lack of systematic theoretical and practical guidance, their opportunities for AI skill development remained limited. They often relied on self-directed learning or fragmented resources, making it difficult to form a systematic skill and knowledge framework, a gap that partly constrained the development of CF (Chen and Xiao, 2025; Zhao et al., 2026). When confronted with complex AI-generated information, these students demonstrated critical awareness but lacked sufficient operational experience to flexibly apply and verify such information, which impeded CF growth (Karaoglan Yilmaz et al., 2025). This constrained CF, in turn, limited their capacity for deep innovative thinking and cross-domain knowledge integration, making it difficult to fully convert AI’s potential value into actual IB (Nijstad et al., 2010). Notably, without sustained attention to this subgroup, a prolonged lack of positive and effective AI-use feedback may engender frustration and self-doubt, causing their initially low AIA to gradually rebound and ultimately adversely affecting CF and IB development (Eysenck et al., 2007; Satoto et al., 2025). In contrast, high-AIL students were more proficient in accessing, communicating with, and applying AI tools. They exhibited stronger evaluative thinking and ethical awareness, held more positive AI values, and were able to critically and flexibly use AI tools and their outputs, thereby sustaining momentum for their IB (Wang et al., 2026).

In light of these differences, educational authorities should implement differentiated strategies to ensure that all students benefit equitably from advances in AI and can leverage these technologies for personal growth and social progress (Walter, 2024). For the C1 group, educators should design progressive introductory programs that build foundational AI knowledge and operational skills, foster a comprehensive and objective understanding of AI, gradually alleviate anxiety stemming from uncertainty, and strengthen students’ confidence and competence in using AI. For the C2 group, although these students recognize the practical value of AI tools, their limited operational proficiency and narrow understanding of AI’s capabilities undermine their confidence in independent use. Structured practical training and case-based learning—such as thematic workshops on AI-assisted writing and data analysis—can help them accumulate successful experiences in authentic tasks, enhance self-efficacy, and support the transition from basic use to proficient application. For the C3 group, educators should look beyond foundational training and provide advanced learning resources, including courses on AI principles, decision-support systems, and big data analytics, while encouraging participation in innovation projects and research practice, thereby leveraging their exemplary roles to deepen the integration of AI technologies and IB (Liu et al., 2023; Walter, 2024; Wang et al., 2026). In summary, with the support of AI, university students’ IB holds tremendous potential. Realizing this potential crucially depends on addressing the accompanying challenges in a responsible and ethical manner. This study suggests that enhancing AI literacy, alleviating AI anxiety, and strengthening cognitive flexibility constitute an effective pathway toward this goal.

### 5.6 Research implications and limitations

At the theoretical level, this study elucidates how AIL fosters university students’ IB. The findings indicate that AIL not only directly stimulates IB but also exerts positive effects through multiple indirect pathways. Specifically, AIL helps alleviate students’ AIA, cultivates more positive AI values, and enhances their critical thinking and CF, thereby providing sustained intrinsic motivation for IB. Furthermore, by employing LPA, this study identified three heterogeneous AIL profiles and revealed significant differences in the serial mediation pathways across these profiles. This moves beyond traditional variable-centered approaches, adopting a person-centered perspective to uncover the structural heterogeneity of AIL and its differential impact on IB. In addition, this study integrated affordance theory and validated the pathway through which AIL enhances IB by strengthening students’ perceived affordances of AI tools. Building on this, the findings further suggest that perceived affordances of AI may themselves be modulated by users’ affective states and cognitive capacities—AIA may attenuate the detection of technological affordances, whereas CF may facilitate their fuller perception and utilization. This offers a promising psychological perspective, worthy of further empirical investigation, for understanding the dynamic generation of affordances in human-AI collaboration. At the practical level, these findings offer actionable implications for educators and administrators. First, while cultivating students’ IB, higher education institutions should also prioritize enhancing their AI-related knowledge and skills. Relevant courses can help students develop a comprehensive and objective understanding of AI technologies, thereby alleviating AIA, enabling them to adapt to technological change with greater flexibility, and promoting innovative thinking. Second, based on the three latent profiles identified, educators should implement differentiated intervention strategies. For students in the low AIL profile, foundational skills training and psychological scaffolding should be provided to help them build basic AI awareness and confidence. For the moderate AIL profile, practical training and case-based instruction can be intensified to facilitate their transition from basic use to proficient application. For the high AIL profile, advanced courses and innovation project opportunities should be offered to fully leverage their leading and exemplary roles. Such precision education strategies can enhance the efficiency of educational resource allocation and ensure that all students benefit equitably from the advancement of AI technologies.

Despite the theoretical and practical insights gained, several limitations should be acknowledged. First, the cross-sectional design precludes causal inferences. Although the serial mediation model was theoretically grounded, the data cannot verify causal direction. Future research could adopt longitudinal or experimental designs to test the proposed causal chain more rigorously. Second, the sample consisted solely of Chinese university students, which may limit the generalizability of the findings to other cultural and educational contexts. Cross-cultural comparisons and replications at different educational stages are warranted. Third, only AIA and CF were examined as mediators; other potential mediating or moderating variables remain unexplored. Future studies could extend the model by incorporating additional psychological and social factors. Fourth, all data were collected through self-report measures, which are prone to social desirability and common method bias. Although statistical checks indicated no serious common method bias, future research could employ multi-source data (e.g., peer ratings, teacher evaluations) or objective indicators (e.g., AI usage logs, innovative product assessments) to strengthen the robustness of the findings. Fifth, the use of convenience sampling via an online survey platform may limit sample representativeness and introduce selection bias. Although participants were recruited from multiple provinces, the non-probability sampling method constrains the generalizability of the results to the broader university student population. Future studies should consider probability sampling strategies, such as stratified random sampling.

## 6 Conclusion

By adopting a latent profile-based serial mediation model and drawing on affordance theory, this study examined how AIL relates to university students’ IB. The results showed that AIL was directly and positively associated with IB, and also indirectly linked to IB through the independent and serial mediating roles of AIA and CF. Three heterogeneous latent profiles of AIL—low, moderate, and high—were identified, and significant differences emerged in the serial mediation pathways across these profiles. These findings suggest that treating AIL solely as a continuous variable may mask meaningful heterogeneity within the population. Accordingly, differentiated educational interventions tailored to students’ latent profiles may more effectively support the development of their IB.

## Data Availability

The original contributions presented in the study are included in the article/Supplementary material, further inquiries can be directed to the corresponding author.

## Footnotes

1
www.sojump.com
