# Supplementary material for: How does artificial intelligence literacy affect university students’ innovative behavior? A serial mediation analysis based on latent profiles
Source: Front Psychol. 2026 Jun 11;17:1844997. doi: 10.3389/fpsyg.2026.1844997 (PMC13293852; doi:10.3389/fpsyg.2026.1844997)
Supplement: Supplementary file 1 [file Table_1.DOCX]

| Table 1. AVE and CR Results for the AIL Scale | | |
| --- | --- | --- |
| Factor | AVE | CR |
| Factor1 | 0.683 | 0.866 |
| Factor2 | 0.692 | 0.947 |
| Factor3 | 0.687 | 0.929 |
| Factor4 | 0.695 | 0.948 |

**Note:** AVE = Average Variance Extracted, CR = Composite Reliability. Factor 1: Knowledge, Factor 2: Skills, Factor 3: Values, Factor 4: Ethics.

The AVE of each dimension of the AIL scale ranged from 0.683 to 0.695, and the CR ranged from 0.866 to 0.948, all exceeding the recommended thresholds of 0.50 and 0.70(Fornell and Larcker, 1981), indicating good convergent validity.

Table 2. Discriminant Validity of the AIL Scale Model

| **No.** | **Model** | **χ²** | **df** | **χ²/df** | **NFI** | **CFI** | **RMSEA** | **Model Comparison** | **∆χ²** | **∆df** |
| --- | --- | --- | --- | --- | --- | --- | --- | --- | --- | --- |
| 1 | Baseline Model | 683.665 | 269 | 2.542 | 0.973 | 0.984 | 0.039 |  |  |  |
| 2 | Three-factor Model 1 | 777.979 | 272 | 2.860 | 0.970 | 0.980 | 0.042 | 2 vs 1 | 94.314*** | 3 |
| 3 | Three-factor Model 2 | 968.031 | 272 | 3.559 | 0.962 | 0.973 | 0.050 | 3 vs 1 | 284.366*** | 3 |
| 4 | Three-factor Model 3 | 1393.516 | 272 | 5.123 | 0.946 | 0.956 | 0.063 | 4 vs 1 | 709.851*** | 3 |
| 5 | Two-factor Model | 920.356 | 274 | 3.359 | 0.964 | 0.975 | 0.048 | 5 vs 1 | 236.691*** | 5 |
| 6 | One-factor Model | 1594.199 | 275 | 5.797 | 0.938 | 0.948 | 0.068 | 6 vs 1 | 910.534*** | 6 |

**Note:** Three-factor Model 1：F1+F2, F3, F4; Three-factor Model 2: F1, F2+F3, F4; Three-factor Model 3: F1, F3, F2+F4; Two-factor Model: F1+F2, F3+F4; One-factor Model:F1+F2+F3+F4. **P* < 0.05 ***P* < 0.01 ****P* < 0.001.

This study examined the discriminant validity of the AIL scale by comparing the baseline four-factor model with five competing models(Anderson and Gerbing, 1988). The results showed that the fit of all competing models was significantly worse than that of the baseline model (*P* < 0.001), indicating good discriminant validity among the four dimensions.

| Table 3. AVE and CR Results for the AIA Scale | | |
| --- | --- | --- |
| Factor | AVE | CR |
| Factor1 | 0.652 | 0.849 |
| Factor2 | 0.636 | 0.840 |
| Factor3 | 0.606 | 0.822 |
| Factor4 | 0.608 | 0.823 |

**Note:** AVE = Average Variance Extracted, CR = Composite Reliability. Factor 1: Learning Anxiety, Factor 2: Job Replacement Anxiety, Factor 3: Socio-technical Blindness, Factor 4: AI Configuration Anxiety.

The AVE of each dimension of the AIA scale ranged from 0.608 to 0.652, and the CR ranged from 0.822 to 0.849, all exceeding the recommended thresholds of 0.50 and 0.70, indicating good convergent validity.

Table 4. Discriminant Validity of the AIA Scale Model

| **No.** | **Model** | **χ²** | **df** | **χ²/df** | **NFI** | **CFI** | **RMSEA** | **Model Comparison** | **∆χ²** | **∆df** |
| --- | --- | --- | --- | --- | --- | --- | --- | --- | --- | --- |
| 1 | Baseline Model | 82.923 | 48 | 1.728 | 0.990 | 0.996 | 0.026 |  |  |  |
| 2 | Three-factor Model 1 | 129.565 | 51 | 2.540 | 0.984 | 0.990 | 0.039 | 2 vs 1 | 46.642*** | 3 |
| 3 | Three-factor Model 2 | 530.703 | 51 | 10.406 | 0.933 | 0.939 | 0.095 | 3 vs 1 | 447.78*** | 3 |
| 4 | Three-factor Model 3 | 929.236 | 51 | 18.220 | 0.883 | 0.888 | 0.129 | 4 vs 1 | 846.313*** | 3 |
| 5 | Two-factor Model | 393.541 | 53 | 7.425 | 0.950 | 0.957 | 0.079 | 5 vs 1 | 310.618*** | 5 |
| 6 | One-factor Model | 1195.075 | 54 | 22.131 | 0.849 | 0.855 | 0.143 | 6 vs 1 | 1112.152*** | 6 |

Compared with the baseline model, the fit of all competing models of the AIA scale was significantly worse (*P* < 0.001), indicating good discriminant validity among the four dimensions.

| Table 5. AVE and CR Results for the CF Scale | | |
| --- | --- | --- |
| Factor | AVE | CR |
| Factor1 | 0.493 | 0.853 |

The AVE of the CF scale was 0.493, slightly below the recommended threshold of 0.50, while its CR reached 0.853. Taken together, the convergent validity of this scale remains at an acceptable level.

| Table 6. AVE and CR Results for the IB Scale | | |
| --- | --- | --- |
| Factor | AVE | CR |
| Factor1 | 0.640 | 0.898 |

The AVE of the IB scale was 0.640 and the CR was 0.898, both exceeding the recommended thresholds, indicating good convergent validity.

Table 7. Confirmatory Factor Analysis-Based Single-Factor Common Method Bias Test

| **Model** | **χ²/df** | **NFI** | **CFI** | **RMSEA** |
| --- | --- | --- | --- | --- |
| One-factor Model | 5.739 | 0.859 | 0.880 | 0.068 |
| AIL Scale | 2.542 | 0.973 | 0.984 | 0.039 |
| AIA Scale | 1.728 | 0.990 | 0.996 | 0.026 |
| IB Scale | 1.817 | 0.995 | 0.998 | 0.028 |
| CF Scale | 1.025 | 0.989 | 1.000 | 0.005 |

To further rule out common method bias, we conducted Harman's single-factor test via confirmatory factor analysis(Podsakoff et al., 2003). All items from all scales were simultaneously loaded onto a single common method factor. This single-factor model demonstrated very poor fit (χ²/df = 5.739, CFI = 0.880), falling far below acceptable thresholds. In contrast, the multi-factor models for the individual scales all showed excellent fit (CFI > 0.98). These results indicate that no single method factor can explain the majority of the variance, and common method bias does not pose a serious threat to the findings of this study.

References

Anderson, J. C., and Gerbing, D. W. (1988). Structural equation modeling in practice: A review and recommended two-step approach. *Psychological bulletin* 103, 411. doi: 10.1037/0033-2909.103.3.411

Fornell, C., and Larcker, D. F. (1981). Evaluating Structural Equation Models with Unobservable Variables and Measurement Error. *Journal of Marketing Research* 18, 39. doi: 10.2307/3151312

Podsakoff, P. M., MacKenzie, S. B., Lee, J.-Y., and Podsakoff, N. P. (2003). Common method biases in behavioral research: A critical review of the literature and recommended remedies. *Journal of Applied Psychology* 88, 879–903. doi: 10.1037/0021-9010.88.5.879
